# Choosing Safe Fish: Too Little Data on the Menu

**Published:** 2005-03

**Authors:** Ernie Hood

In recent years, both the benefits and the potential health risks of eating certain types of fish have been well publicized. Consumption advisories by government agencies as well as media reports have raised public awareness of high mercury concentrations in certain fish, particularly swordfish, shark, king mackerel, and tilefish. In this month’s issue, scientists at Rutgers University, the Environmental and Occupational Health Sciences Institute, and the New Jersey Department of Environmental Protection contend that such efforts still are not enough to allow consumers to make truly informed decisions about which fish to eat, how often, and in what quantities **[*EHP*** **113:266–271]**.

With the overall objective of exploring how the information communicated in public health advisories might be enhanced, the group looked at the potential variation in fish mercury levels between regions within a state, between neighborhoods of different economic strata, and between types of stores. They also wanted to determine whether regional levels were significantly different from reported national levels posted by the Food and Drug Administration (FDA) online at http://www.cfsan.fda.gov/~frf/sea-mehg.html.

For the statewide comparison, the investigators analyzed the mercury content of samples of three types of fish commonly available in New Jersey—tuna, flounder, and bluefish. The fish were purchased between July and October 2003 from grocery stores and fish markets in regions throughout the state, and in both high- and low-income communities.

The species—as expected—varied significantly in their mercury content, with large predatory tuna containing the most and bottom-feeding flounder the least. Mercury content did not vary significantly among store types or economic areas. Just one regional difference emerged: flounder purchased at fish markets along the Jersey shore had higher mercury levels than flounder from markets in other areas, possibly due to the fish coming into the stores from different sources, such as regional distribution centers.

To compare actual mercury measures against data reported by the FDA, the team purchased and assayed samples of six additional types of fish (Chilean sea bass, porgy, red snapper, croaker, cod, and whiting) and two types of shellfish (shrimp and scallops) from central New Jersey markets. These species were chosen because of their wide availability in the state.

Mean levels of mercury were higher in the sea bass, croaker, whiting, and shrimp available in New Jersey—as well as the tuna sampled in the first tier of the study—than predicted by the FDA’s data; the actual mean for one fish, croaker, was nearly three times the FDA estimate. The authors say these discrepancies show that the FDA should update its database (the data provided were collected mainly from 1990 to 1992). They also suggest that the agency consider providing regional breakdowns of aggregate mercury levels so state agencies can evaluate possible risks for their citizens.

According to the researchers, this was the first study of mercury levels in commercial fish that included examination of fish availability, cost, and consumer preference as variables in consumer choice. Flounder struck the best balance between ready availability, affordable cost, and low mercury content. The authors suggest that state agencies responsible for health risk communication conduct more comprehensive studies and disseminate findings on consumers’ fish preferences as well as fish cost, availability, and contamination, including data on commercial fish species with low levels of mercury. Such information would enhance consumers’ ability to make rational decisions about fish consumption.

Fish clearly can be a healthful food providing a relatively low-fat protein source as well as beneficial nutrients that protect against cardiovascular disease, the authors point out. They are pursuing studies that more clearly portray the balance between benefits and risks associated with eating different types of fish.

## Figures and Tables

**Figure:**
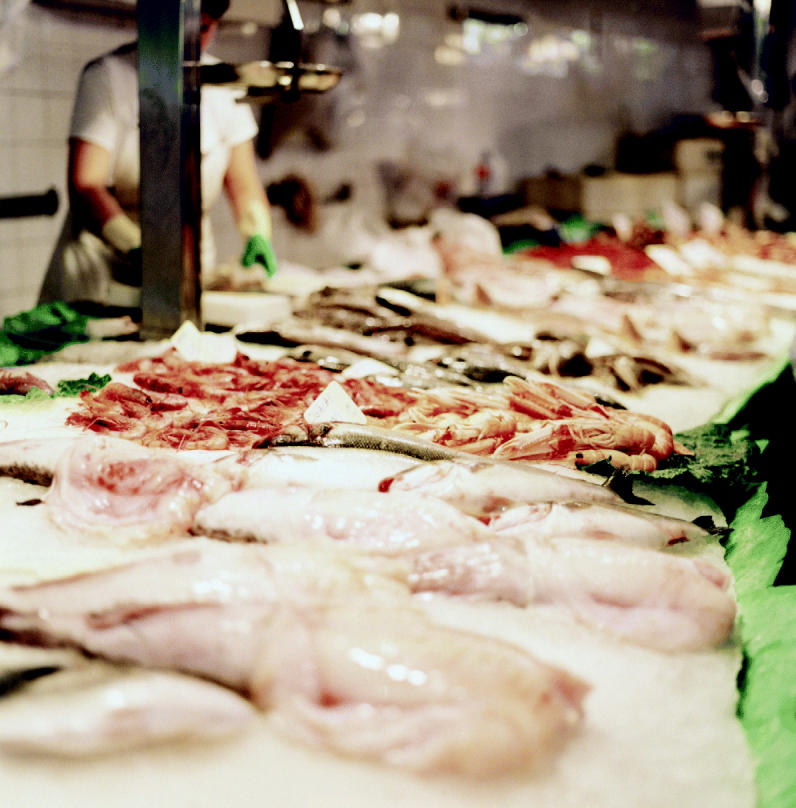
**Buyer beware.** Researchers say consumers’ ability to choose the safest seafood is hampered by a lack of reliable, accurate data on mercury levels in locally sold fish.

